# Supplementary material for: B38-CAP is a bacteria-derived ACE2-like enzyme that suppresses hypertension and cardiac dysfunction
Source: Nat Commun. 2020 Feb 26;11:1058. doi: 10.1038/s41467-020-14867-z (PMC7044196; doi:10.1038/s41467-020-14867-z)
Supplement: Supplementary file 1 — Supplementary Information [file 41467_2020_14867_MOESM1_ESM.pdf]

Supplementary information

B38-CAP is a bacteria-derived ACE2-like enzyme that suppresses hypertension and cardiac dysfunction.

Minato, et al.

Supplementary Table 1. Accession codes of DNA or amino acid sequences

Enzyme	Origin	UniProt	DDBJ/EMBL /GeneBank	PDB
B38-CAP	*Paenibacillus* sp. B38		LC406946	
BS-CAP	*Bacillus subtilis* subsp. *subtilis* str. 168	P50848	L47838	3HQ2
BA-CAP	*Bacillus amyloliquefaciens* NBRC 3022		LC417450	
Human ACE2	*Homo sapiens*	Q9BYF1	AB193259	1R4L
Mouse ACE2	*Mus musculus*	Q8R010	AB053182	
Rat ACE2	*Rattus norvegicus*	Q5EGZ1	AY881244	
Fly ACER	*Drosophila melanogaster*	Q9VLJ6		

Supplementary Table 2. Echocardiographic and physical parameters in mice co-infused with Ang II and B38-CAP for 2 weeks

	vehicle			vehicle + B38-CAP			Ang II + vehicle			Ang II + B38-CAP		
N		10			11			11			11	
age (weeks)		9			9			9			9	
BW (g)	20.53	±	1.62	23.92	±	1.53	22.24	±	1.26	22.38	±	1.23
HR (bpm)	561	±	60	562	±	61	579	±	49	564	±	48
FS (%)	52.45	±	4.42	52.22	±	2.66	45.88	±	7.15*	51.50	±	4.28#
EF (%)	84.25	±	3.82	84.16	±	2.47	77.62	±	7.44*	83.55	±	3.60#
LVESD (mm)	1.55	±	0.23	1.59	±	0.24	1.70	±	0.32	1.51	±	0.20
LVEDD (mm)	3.26	±	0.44	3.32	±	0.38	3.11	±	0.25	3.12	±	0.25
IVSD (mm)	0.68	±	0.06	0.72	±	0.04	0.94	±	0.05***	0.75	±	0.05###
PWD (mm)	0.69	±	0.06	0.70	±	0.03	0.93	±	0.05***	0.79	±	0.06###
HW / BW (mg/g)	5.00	±	0.23	4.54	±	0.19	6.38	±	0.35***	5.48	±	0.24###
HW / TL (mg/mm)	4.73	±	0.44	4.89	±	0.18	6.47	±	0.38***	5.44	±	0.23###
LW / BW (mg/g)	5.71	±	0.29	5.21	±	0.33	5.67	±	0.26	5.51	±	0.35
LW / TL (mg/mm)	5.39	±	0.32	5.62	±	0.48	5.75	±	0.36	5.46	±	0.25

Results are presented as mean ± s.e.m. One-way ANOVA plus Sidak's multiple-comparisons test was used to detect significance. *P < 0.01 versus vehicle; ***P < 0.0001 versus vehicle; # P < 0.05 versus Ang II + vehicle; ###P < 0.0001 versus Ang II + vehicle.

Abbreviations; BW, body weight; HR, heart rate; FS, left ventricular fractional shortening; EF, left ventricular ejection fraction; LVESD, left ventricular end-systolic diameter; LVEDD, left ventricular end-diastolic diameter; IVSD, end-diastolic interventricular septal wall thickness; PWD, left ventricular end-diastolic posterior wall; HW, heart weight; TL, tibia length; LW, lung weig

Supplementary Table 3. Echocardiographic and physical parameters in the Ang II- infused mice with daily injection of B38-CAP for 4 weeks

	vehicle	vehicle + B38-CAP	Ang II + vehicle	Ang II + B38-CAP
N	9	5	5	5
age (weeks)	9	9	9	9
BW (g)	23.54 ± 1.28	24.98 ± 2.02	25.34 ± 1.49	24.80 ± 0.74
HR (bpm)	594 ± 35	596 ± 41	605 ± 31	646 ± 15
FS (%)	43.93 ± 8.58	51.86 ± 3.64	37.47 ± 5.90***	51.82 ± 2.58###
EF (%)	72.48 ± 9.83	84.15 ± 3.54	68.23 ± 7.65	83.73 ± 2.44#
LVESD (mm)	1.78 ± 0.37	1.41 ± 0.25	2.00 ± 0.33	1.57 ± 0.14
LVEDD (mm)	3.15 ± 0.28	2.91 ± 0.29	3.17 ± 0.03	3.24 ± 0.24
IVSD (mm)	0.86 ± 0.12	0.76 ± 0.01	0.96 ± 0.04***	0.66 ± 0.05###
PWD (mm)	0.86 ± 0.13	0.70 ± 0.03	0.98 ± 0.03***	0.71 ± 0.04###
HW / BW (mg/g)	4.86 ± 0.20	4.72 ± 0.14	7.04 ± 0.46***	5.26 ± 0.46###
HW / TL (mg/mm)	5.13 ± 0.25	4.85 ± 0.32	7.73 ± 0.50***	5.78 ± 0.59###
LW / BW (mg/g)	5.28 ± 0.29	5.36 ± 0.55	6.98 ± 0.15***	6.39 ± 0.83
LW / TL (mg/mm)	5.46 ± 0.25	4.85 ± 0.32	7.68 ± 0.42***	7.01 ± 0.93

Results are presented as mean ± s.e.m. One-way ANOVA plus Sidak's multiple-comparisons test was used to detect significance. ***P < 0.0001 versus vehicle; # P < 0.05 versus Ang II + vehicle; ###P < 0.0001 versus Ang II + vehicle.

Abbreviations; BW, body weight; HR, heart rate; FS, left ventricular fractional shortening; EF, left ventricular ejection fraction; LVESD, left ventricular end-systolic diameter; LVEDD, left ventricular end-diastolic diameter; IVSD, end-diastolic interventricular septal wall thickness; PWD, left ventricular end-diastolic posterior wall; HW, heart weight; TL, tibia length; LW, lung weight.

Supplementary Table 4. Echocardiographic and physical parameters in the mice under TAC infused with B38-CAP for 2 weeks

	sham + vehicle			sham + B38-CAP			TAC + vehicle			TAC + B38-CAP		
N		7			5			9			8	
age (wks)		10			10			10			10	
Pre-TAC BW (g)	25.08	±	2.59	24.00	±	0.97	26.94	±	0.61	26.80	±	0.46
Post-TAC BW (g)	26.00	±	2.55	24.85	±	0.97	25.26	±	1.52†	26.68	±	1.42
HR (bpm)	586	±	56	594	±	57	580	±	29	590	±	26
FS (%)	49.24	±	5.24	51.57	±	6.78	25.26	±	7.00***	50.93	±	3.18####
EF (%)	81.14	±	4.54	83.24	±	5.59	45.06	±	9.28***	83.18	±	2.92####
LVESD (mm)	1.71	±	0.20	1.43	±	0.23	2.87	±	0.33***	1.50	±	0.26####
LVEDD (mm)	3.37	±	0.15	2.96	±	0.22	3.71	±	0.03	3.03	±	0.38####
IVSD (mm)	0.69	±	0.04	0.67	±	0.06	1.00	±	0.03***	0.78	±	0.03####
PWD (mm)	0.69	±	0.03	0.70	±	0.04	1.00	±	0.03***	0.86	±	0.05####
HW / BW (mg/g)	4.91	±	0.43	4.82	±	0.32	8.43	±	1.05***	5.33	±	0.50####
HW / TL (mg/mm)	5.55	±	0.39	5.20	±	0.17	8.96	±	0.84***	6.12	±	0.67####
LW / BW (mg/g)	5.60	±	0.68	5.50	±	0.44	10.11	±	4.41**	5.45	±	0.45##
LW / TL (mg/mm)	6.33	±	0.74	5.93	±	0.34	10.63	±	4.18**	6.26	±	0.67##

Results are presented as mean ± s.e.m. Two-tailed unpaired Student's t-test was used to detect significance of BW transition. One-way ANOVA plus Sidak's multiple-comparisons test was used to detect significance except for BW transition. †P < 0.05 versus Pre-TAC BW TAC + vehicle; **P < 0.001 versus sham + vehicle; ***P < 0.0001 versus sham + vehicle; ##P < 0.001 versus TAC + vehicle, ####P < 0.0001 versus TAC + vehicle. Abbreviations; BW, body weight; HR, heart rate; FS, left ventricular fractional shortening; EF, left ventricular ejection fraction; LVESD, left ventricular end-systolic diameter; LVEDD, left ventricular end-diastolic diameter; IVSD, end-diastolic interventricular septal wall thickness; PWD, left ventricular end-diastolic posterior wall; HW, heart weight; TL, tibia length; LW, lung weight.

Supplementary Table5. Echocardiographic parameters in the C57BL/6N mice with established cardiac dysfunction treated with B38-CAP for 2 weeks

	sham + vehicle before treatment	sham + vehicle after treatment	TAC + vehicle before treatment	TAC + vehicle after treatment	TAC + B38-CAP before treatment	TAC + B38-CAP after treatment
N	5	5	8	8	8	8
age (wks)	12	14	12	14	12	14
BW	24.86 ± 0.80	27.44 ± 1.06	24.68 ± 1.10	28.13 ± 1.56	25.31 ± 0.79	27.68 ± 0.77
HR (bpm)	607 ± 31	609 ± 28	641 ± 37	634 ± 53	588 ± 59	604 ± 39
FS (%)	52.20 ± 2.29	55.53 ± 2.79	28.91 ± 2.35***	20.07 ± 3.55	26.71 ± 2.93***	31.27 ± 2.62$^{\#\#\# \dagger}$
EF (%)	83.99 ± 1.93	87.03 ± 1.99	56.47 ± 3.66***	41.39 ± 6.26	52.73 ± 4.71***	59.67 ± 4.05$^{\#\#\# \dagger}$
LVESD (mm)	1.51 ± 0.17	1.41 ± 0.21	2.49 ± 0.24***	3.18 ± 0.39	2.80 ± 0.14***	2.28 ± 0.14$^{\#\#\# \dagger\dagger\dagger}$
LVEDD (mm)	3.16 ± 0.29	3.31 ± 0.41	3.50 ± 0.30	3.97 ± 0.38	3.75 ± 0.15	3.41 ± 0.28$^{\#\#}$
IVSD (mm)	0.70 ± 0.03	0.73 ± 0.02	1.02 ± 0.05***	1.06 ± 0.07	1.03 ± 0.08***	0.94 ± 0.05$^{\#\# \dagger}$
PWD (mm)	0.72 ± 0.02	0.72 ± 0.06	1.01 ± 0.05***	1.06 ± 0.08	0.96 ± 0.07***	0.97 ± 0.08$^{\#}$

Results are presented as mean ± s.e.m. One-way ANOVA plus Sidak's multiple-comparisons test was used to detect significance. *P < 0.05 versus sham + vehicle before treatment; **P < 0.001 versus sham + vehicle before treatment; ***P < 0.0001 versus sham + vehicle before treatment; $^{\#}$P < 0.05 versus TAC + vehicle after treatment; $^{\#\#}$P < 0.001 versus TAC + vehicle after treatment; $^{\#\#\#}$P < 0.0001 versus TAC + vehicle after treatment. Two-tailed paired t-test was used to detect significance. †P < 0.05 versus TAC + B38-CAP before treatment; ††P < 0.001 versus TAC + B38-CAP before treatment; †††P < 0.0001 versus TAC + B38-CAP before treatment.

Abbreviations; BW, body weight; HR, heart rate; FS, left ventricular fractional shortening; EF, left ventricular ejection fraction; LVESD, left ventricular end-systolic diameter; LVEDD, left ventricular end-diastolic diameter; IVSD, end-diastolic interventricular septal wall thickness; PWD, left ventricular end-diastolic posterior

Supplementary Table 6. Primer sequences for qRT-PCR

Gene	Primers	Sequence (5'-3')
BNP	Forward	ATGGATCTCCTGAAGGTGCTG
	Reverse	GTGCTGCCTTGAGACCGAA
Myh7	Forward	GCAGCAGTTGGATGAGCGAC
	Reverse	TGCCTCCTCCAGCCTTTCAC
Tgfb2	Forward	AGAAGCGCGCTTTGGATGCTGC
	Reverse	TGGGACACACAGCAAGGGGAAG
Postn	Forward	TGCTCTGCTGCTGCTGTTCCTG
	Reverse	TGCTGGAGGGCACAGACGTTTG
Col8a	Forward	TCAGACTCATTCAGGCCGGTGC
	Reverse	CGCGCAAACTGGCTAACGGTAC
Gapdh	Forward	CTGCACCACCAACTGCTTAG
	Reverse	GTCTTCTGGGTGGCAGTGAT

B38-CAP: Carboxypeptidase from *Paenibacillus* sp B38
BS-CAP: Carboxypeptidase from *Bacillus subtilis* subsp. *subtilis*
BA-CAP: Carboxypeptidase from *Bacillus amyloliquefaciens*

```
B38-CAP   1:MTVHNSKALAPFLETVKKLKAYGEALGVLYWDLRTGAPRKGMDMRSEVIGSLSGDMFKLSTSPELGEWLSELEQPETFGKLSEIERKLVT 90
BS-CAP    1:--MEIHTYEKEFFDLLKRISHYSEAVALMHWDSRTGAPKNGSEDRAESIGQLSTDIFNIQTSDRMKELIDVLYERFD--DLSEDTKKAVE 86
BA-CAP    1:--MDLHTYEKEFFDLLKRVSHYVEAIALMHWDLRTGAPKKGSDDRAESIGQLSADVFQIQTSDRMKELIDILLAHAE--ELPEDTVKAAE 86
                       *    *    * **     ** *****  *   * * ** ** * *   **    *    *        * *   *

B38-CAP  91:DTREEYDRSVKIPPKLYEEHVVICSQAESKWEEAKAANDYEGFQPYLEKVIDYTQQFIDLWGPKETRYDTLLDQYEPGMTVKELDKVFGG 180
BS-CAP   87:LAKKEYEENKKIPEAEYKEYVILCSKAETAWEEAKGKSDFSLFSPYLEQLIEFNKRFITYWGYQEHPYDALLDLFEPGVTVKVLDQLFAE 176
BA-CAP   87:LAKKDYDHNKKIPEDEYKEYVILTSRAETAWEDAKAASDFSMFAPYLEKLIDFNKRFISYWGYEDHPYNALLDIFEPGVTVKVLDQLFSE 176
                 *    ***   * * *   * **  ** **   *   * ****  *     **  **     *   *** *** *** **  *

B38-CAP 181:LREQLVPLAAAIAASKHQPDTSFLRQNYDKQAQKAFSLRILKQMGYDFEAGRLDESTHPFATGLNTGDVRITTRYLEDDVTSALFGTIHE 270
BS-CAP  177:LKEAIIPLVKQVTASGNKPDTSFITKAFPKEKQKELSLYFLQELGYDFDGGRLDETVHPFATTLNRGDVRVTTRYDEKDFRTAIFGTIHE 266
BA-CAP  177:LKEAIIPLIKKVTESGNEPDTSFITKTFSKEQQRDLSLYFLKEFGYDFDGGRLDETVHPFATTINRGDVRVTTRYDENDFRTAIFGTIHE 266
            * *   **      *   *****      *  *   **  *   ****  *****  *****  * **** **** * *   * ******

B38-CAP 271:GGHALYEQNIMAELDGTTLSTGTSMGIHESQSRFWENVIGRSKPFWQHNFAALQQQFPGQL-DVTLDQFYRGNNVVQPSLIRIEADELTY 359
BS-CAP  267:CGHAIYEQNIDEALSGTNLSDGASMGIHESQSLFYENFIGRNKHFWTPYYKKIQEASPVQFKDISLDDFVRAINESKPSFIRVEADELTY 356
BA-CAP  267:CGHAIYEQNIDEALSGTNLSDGASMGIHESQSLFYENFIARNQHFWTAYYEKMVEASPDQFQDVKREDFVRAVNEAKPTFIRIEADELTY 356
             *** *****   * ** ** * ********* * ** * *   **           * *  *     * *  *   ** *******

B38-CAP 360:NLHIIIRYEIEKMIFNEGAKAADLPAIWNEKYKEYLGIEPPTNAEGVLQDVHWSGGAFGYFPSYSLGNMYAAQFADTLERELPNFWELVS 449
BS-CAP  357:PLHIIIRYEIEKAIFSNEVSVEDLPSLWNQKYQDYLGITPQTDAEGILQDVHWAGGDFGYFPSYALGYMYAAQLKQKMLEDLPEFDALLE 446
BA-CAP  357:PLHIIIRYEIEKAIFNNEVTVEELPALWNQKYHDYLGITPPSDAKGILQDVHWAGGDFGYFPSYALGYMYAAQLKHTMLDDLPEFDQLIE 446
             *********** **        **  ** **  **** *   * * ****** ** ******* ** *****        ** *  *

B38-CAP 450:AGNLLPIKEWLSERIYKYGKLRTPSELIQNVTGKPLDPQYLVKYLEKKYSEIYKL                                    504
BS-CAP  447:RGEFHPIKQWLTEKVHIHGKRKKPLDIIKDATGEELNVRYLIDYLSNKYSNLYLL                                    501
BA-CAP  447:RGDFEPIKQWLTEKVHQHGRRKMPLDIIKDATGEELNVQYLIEYLVGKYSNLYL-                                    500
             *   *** ** *     *    *   *   **  *   **  **  ***  *
```

Supplementary Figure 1. **Amino acid sequence alignment of B38-CAP, BA-CAP and BS-CAP.**

Red boxes indicate metal-binding or catalytic residues, and black boxes indicate substrate-binding residues. Asterisks (*) indicate amino acid residues that are identical among these enzymes.

a Purification of recombinant B38-CAP expressed in *E. coli*.

	Total volume (ml)	Total activity (U)	Total protein (mg)	Specific activity (U/mg)	Purification (-fold)	Yield (%)
Culture medium	920					
Supernatant of cell lysate	43.5	210	224	0.933	1	100
Q-sepharose FF	80.0	126	38.5	3.27	3.50	60.1
Superdex 75pg	21.0	112	15.5	7.26	7.78	53.7

b Purification of rhACE2 expressed in baculovirus-Sf9 insect cells.

	Total volume (ml)	Total activity (U)	Total protein (mg)	Specific activity (U/mg)	Purification (-fold)	Yield (%)
Culture medium	500					
Supernatant	50.0	119	279	0.426	1	100
Ni-NTA affinity column & dialysis	9.8	34.5	2.71	12.7	29.8	29.0

c

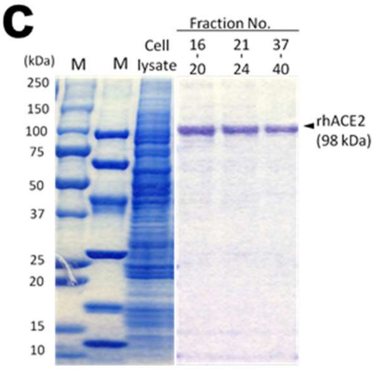

d

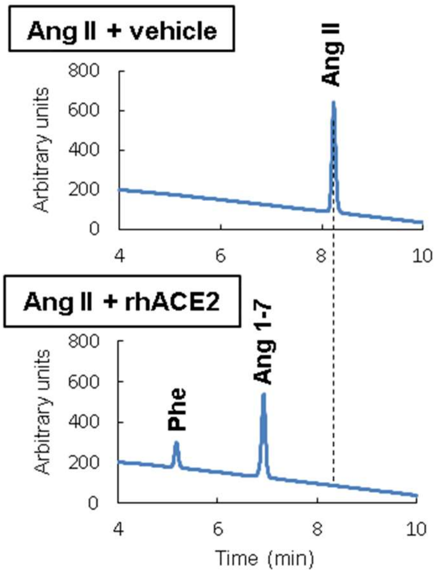

Supplementary Figure 2. **Purification of recombinant proteins of B38-CAP and human ACE2.**

a, Purification of recombinant B38-CAP expressed in *E. coli*. Specific activity was measured with the ACE2 substrate, Nma-His-Pro-Lys(Dnp). **b**, Purification of recombinant human ACE2 (rhACE2) expressed in baculovirus-Sf9 insect cell system. Specific activity was measured with Nma-His-Pro-Lys(Dnp). **c,** SDS-PAGE analysis of purified rhACE2 protein. **d,** HPLC analysis of Ang II peptide treated with rhACE2.

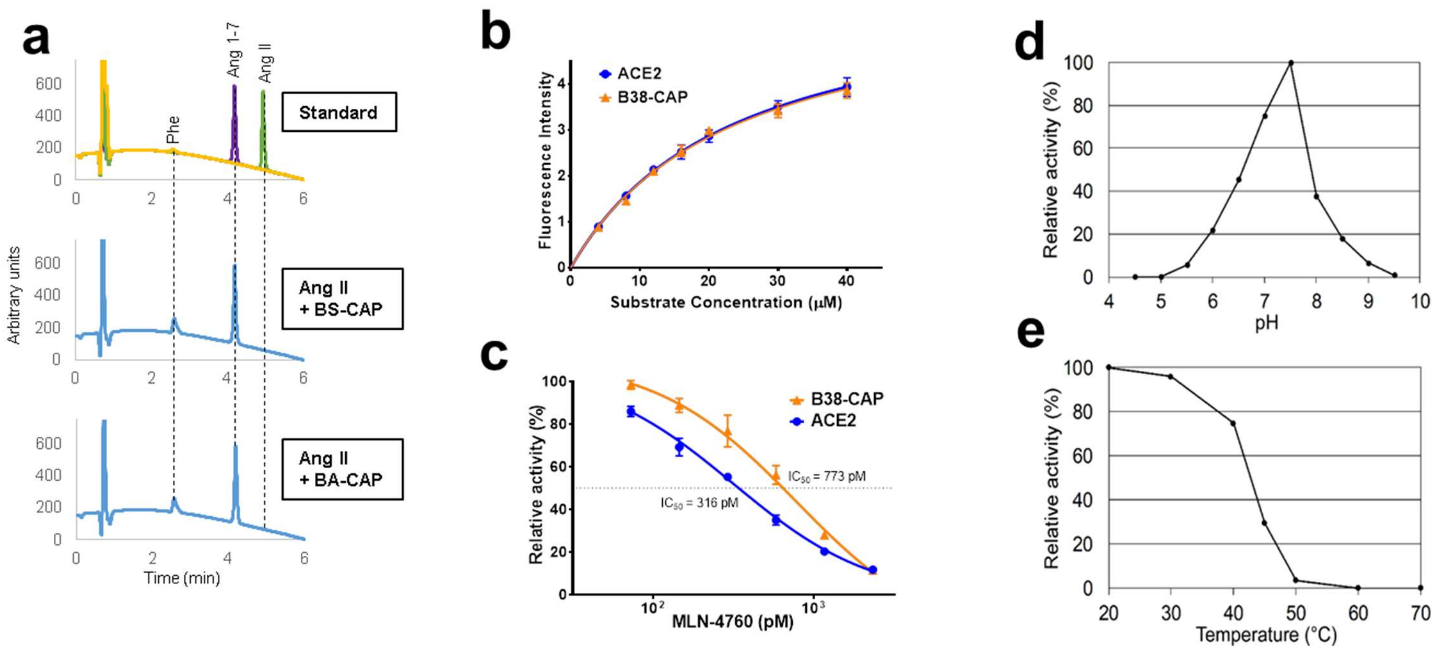

Supplementary Figure 3. **ACE2-like proteolytic activity of BS-CAP, BA-CAP or B38-CAP.**

a, HPLC analysis of Ang II peptide treated with BS-CAP or BA-CAP. Ang II (5 nmol) was incubated with vehicle, BS-CAP or BA-CAP (5 μg each), then subjected to HPLC analysis. **b**, Michaelis-Menten curve for hydrolysis of ACE2 substrate, Nma-His-Pro-Lys(Dnp) by B38-CAP or human ACE2. The values of K_m and k_{cat} were determined and shown in Table 1. **b-c**, Each values are means ± SEM. n = 3 independent experiments. **c**, Titration of MLN-4760, an ACE2 inhibitor on proteolytic activities of B38-CAP or human ACE2. The values of IC_{50} were determined and shown in Table 1. **d-e**, Dependence of ACE2-like proteolytic activity of B38-CAP on pH (**d**) and temperature (**e**). Each result is the mean value for triplicate determinations.

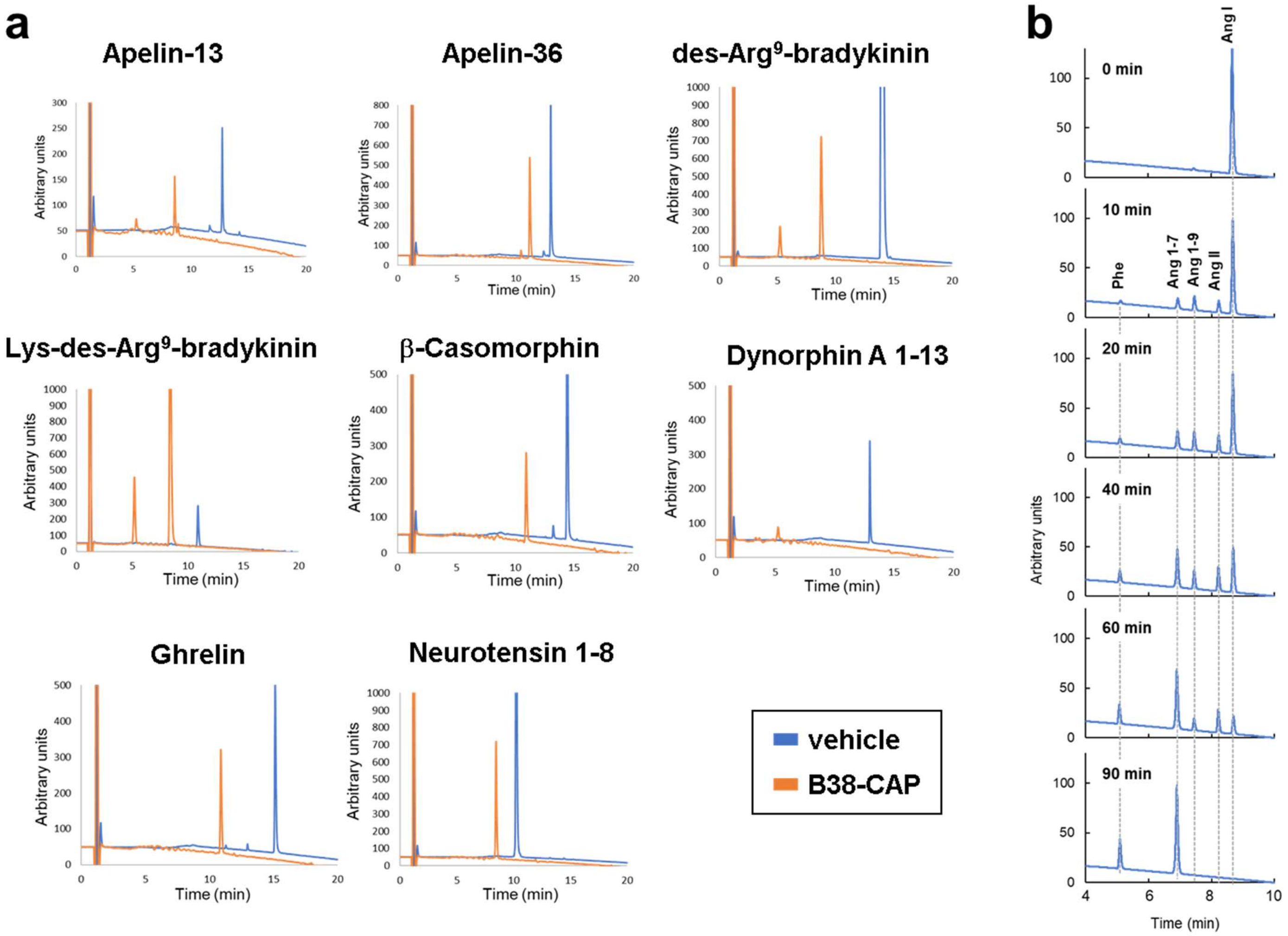

Supplementary Figure 4. **Proteolytic cleavage of ACE2 substrate biological peptides by B38-CAP.**

a, Various ACE2 substrate biological peptides were incubated with vehicle or recombinant B38-CAP protein, then subjected to HPLC analysis. **b,** Kinetic analysis for hydrolysis of Ang I with B38-CAP. HPLC analysis of angiotensin peptides in the time course of reaction.

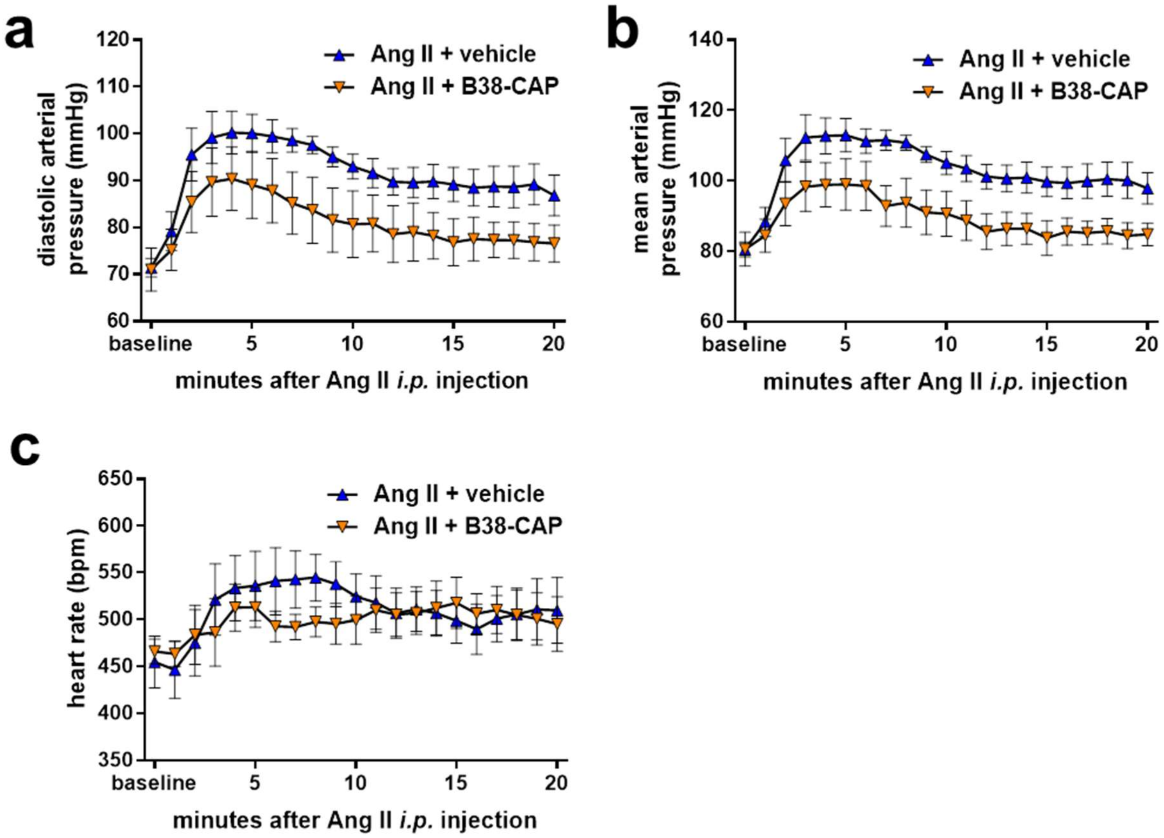

Supplementary Figure 5. **Invasive measurements of arterial blood pressure.**

Diastolic (**a**) and mean (**b**) arterial blood pressure and heart rate (**c**) were measured as shown in Fig. 2b-c (n = 6 per group). All values are means ± SEM. Two-way ANOVA with Sidak's multiple comparisons test.

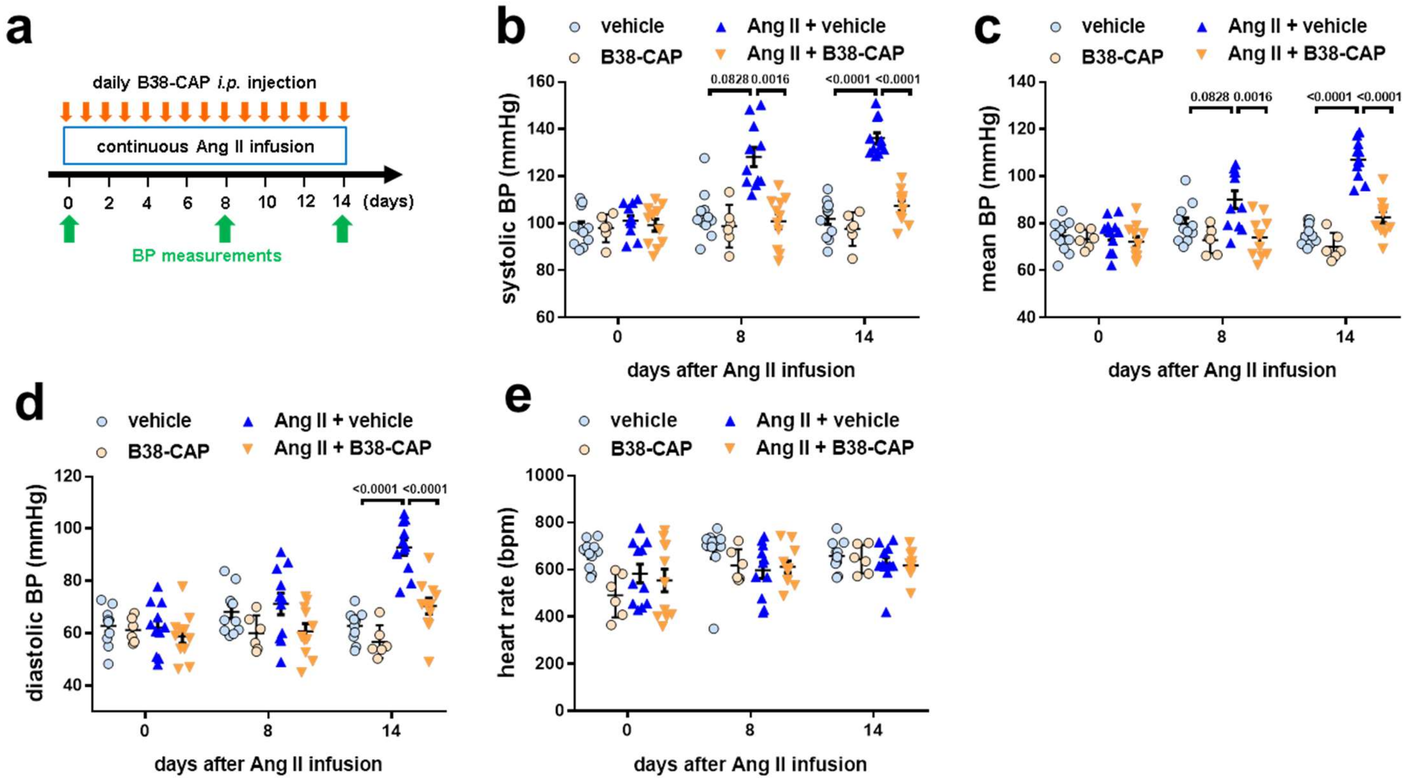

Supplementary Figure 6. **Effects of daily injection of B38-CAP on Ang II-induced hypertension.**

a, Experimental protocol; Ang II (1 mg/kg/day) was continuously infused with osmotic mini-pumps, and the mice were treated with a daily bolus *i.p.* injection of vehicle or B38-CAP (2 mg/kg/day) for 2 weeks. Blood pressure (BP) was measured in conscious mice by tail-cuff system at 2 hours after the last injection. **b-e,** Blood pressure measurements. Systolic (**b**), mean (**c**), diastolic (**d**) blood pressure and heart rate (**e**) were measured at 2 hours after injection. n = 6-11 independent animals. All values are means ± SEM. **b-e**, Two-way ANOVA with Sidak's multiple comparisons test. Numbers above square brackets show significant *P* values. n.s. not significant.

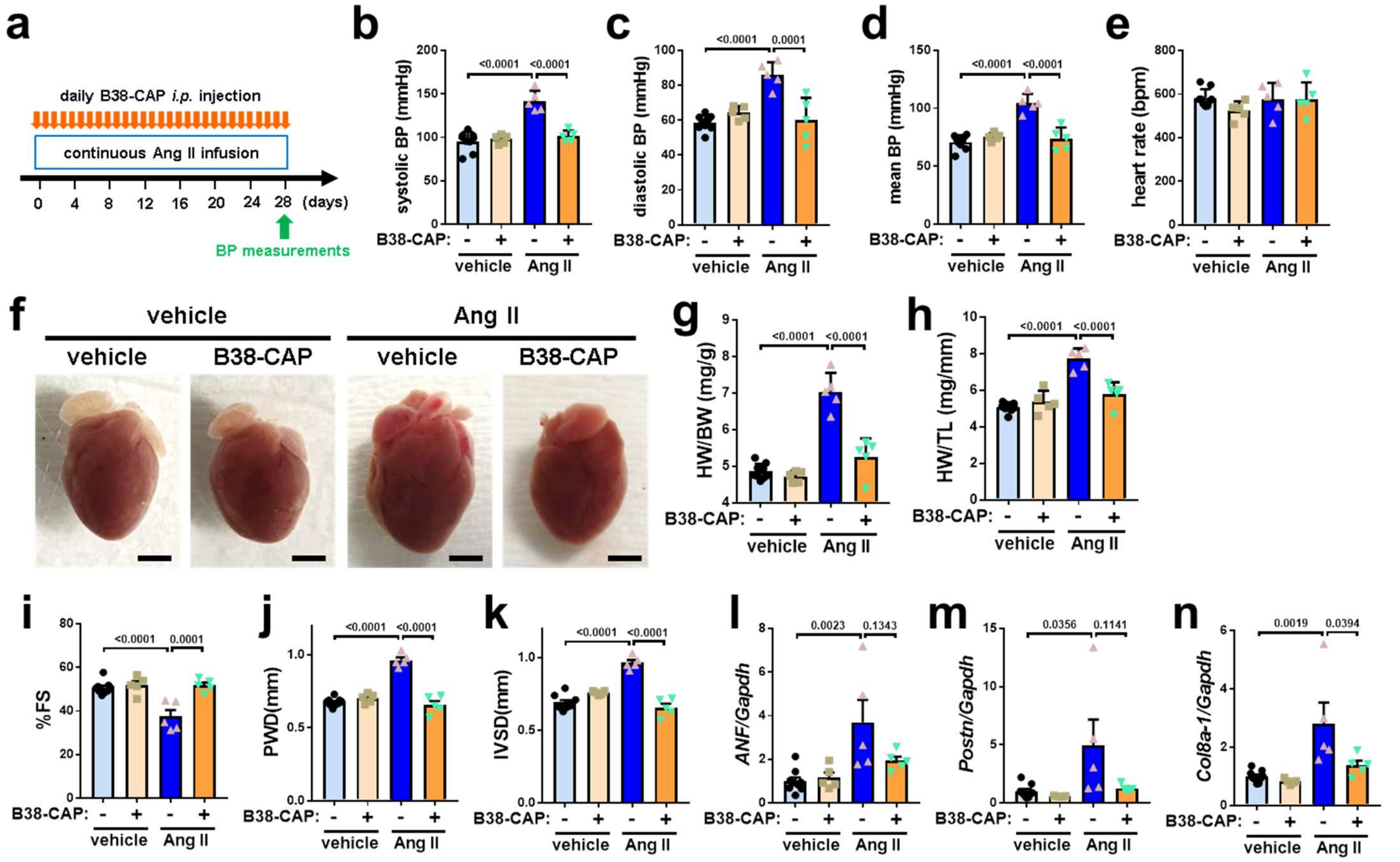

Supplementary Figure 7. Effects of 4 weeks daily injection of B38-CAP on Ang II-induced hypertension and cardiac hypertrophy.

a, Experimental protocol; Ang II-infused mice (1 mg/kg/day) were treated with a bolus injection of B38-CAP (2 mg/kg *i.p.*) per 12 hours for 4 weeks. **b-e**, Blood pressure (BP) measurements. Systolic (**b**), diastolic (**c**), mean (**d**) blood pressure and heart rate (**e**) were measured by tail-cuff system at 2 hours after last injection. **f-h**, Cardiac hypertrophy. Macroscopic heart images (**f**), HW/BW (**g**) and HW/TL (**h**) are shown. Bars indicate 2 mm. **i-k**, Echocardiography parameters of %FS (**i**), PWD (**j**), and IVSD (**k**) in the mouse hearts. Complete echocardiography data are shown in Supplementary Table 3. n = 5-9 independent animals. **l-n**, qRT-PCR analysis of heart failure gene expressions in the hearts; mRNA levels of *ANF* (**l**), *Postn* (**m**) and *Col8a1* (**n**) are shown. All values are means ± SEM. n = 5-9 biologically independent samples. **b-e**, **g-n**, Two-way ANOVA with Sidak's multiple comparisons test. Numbers above square brackets show significant *P* values.

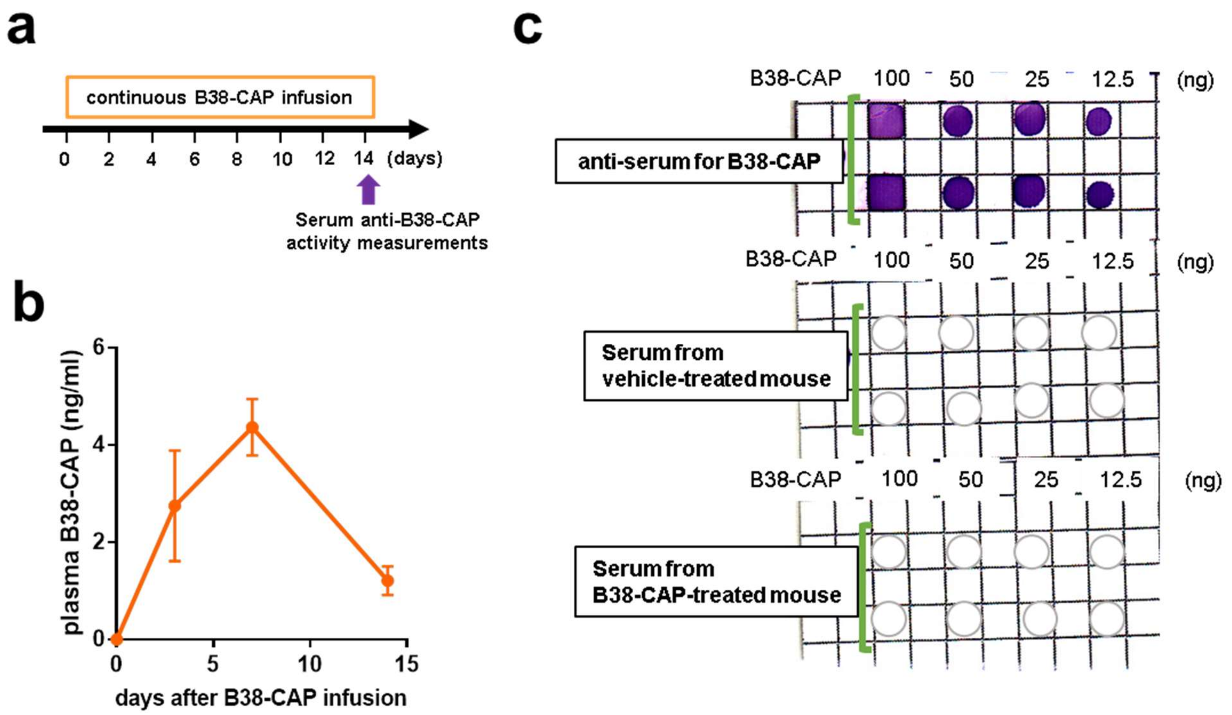

Supplementary Figure 8. B38-CAP levels in the serum of mice continuously infused with B38-CAP.

a, Experimental protocol; The mice were treated with continuous infusion of B38-CAP (2 mg/kg/day). **b,** Plasma B38-CAP levels in mice (n = 6 per group). B38-CAP enzymatic activity was measured by using a B38-CAP-specific substrate, Nma-Leu-Pro-Lys(Dnp). **c,** Dot blot analysis for anti-B38-CAP activity measurements. The membranes spotted with B38-CAP protein were incubated with the serum (x100 dilution) from mice continuously infused with vehicle (*middle*) or B38-CAP (*bottom*). The serum (x100 dilution) from a rabbit immunized with B38-CAP served as a positive control (*top*).

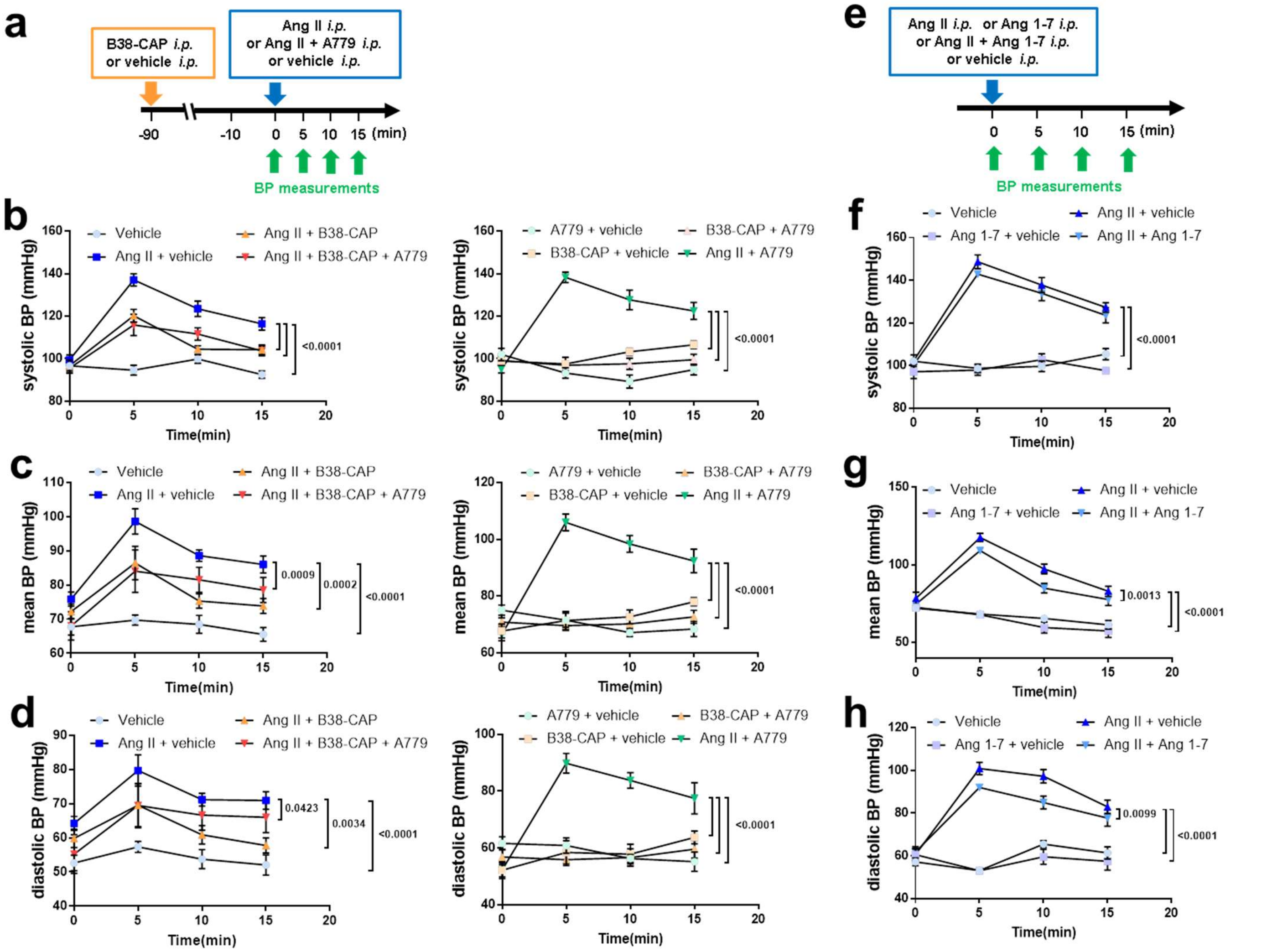

Supplementary Figure 9. No obvious involvement of Ang 1-7 – Mas receptor axis in B38-CAP-mediated hypotensive action.

a-d, No obvious effects of A779, an Mas receptor antagonist, on B38-CAP-mediated hypotensive action. Experimental protocol (**a**); Mice were pretreated with vehicle or B38-CAP (2 mg/kg *i.p.*), and Ang II (0.2 mg/kg *i.p.*), A779 (0.2 mg/kg *i.p.*) or its combination was *i.p.* injected. Systolic (**b**), mean (**c**) and diastolic (**d**) blood pressure were measured every 5 minutes by tail-cuff system under awake condition (n = 6 per group). **e-h**, No obvious effects of Ang 1-7 on Ang II-induced hypertension. Experimental protocol (**e**); Mice were *i.p.* injected with vehicle, Ang II (0.2 mg/kg *i.p.*), Ang 1-7 (0.2 mg/kg *i.p.*) or its combination. Systolic (**f**), mean (**g**) and diastolic (**h**) blood pressure were measured every 5 minutes by tail-cuff system (n = 6 per group). All values are means ± SEM. **b-d**, **f-h**, Two-way ANOVA with Sidak's multiple comparisons test. Numbers next to square brackets show significant *P* values.

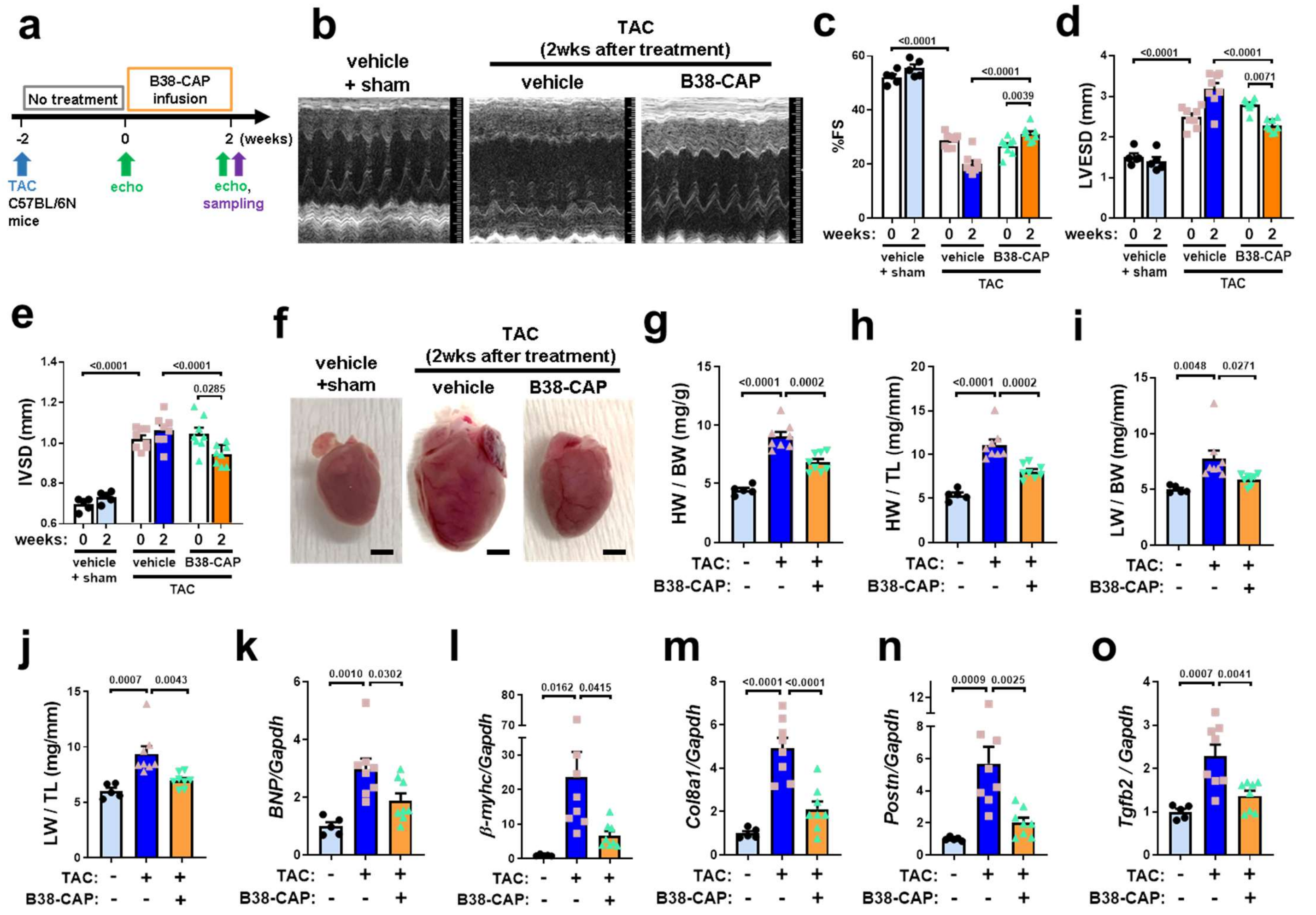

Supplementary Figure 10. Therapeutic effects of B38-CAP on established severe cardiac dysfunction in C57BL/6N mice.

Experimental protocol (**a**); The C57BL/6N mice had TAC surgery at 2 weeks before treatment, and B38-CAP (2 mg/kg/day) or vehicle was continuously infused with osmotic mini-pumps. Representative M-mode echocardiography images (**b**). Echocardiography parameters of %FS (**c**), LVESD (**d**) and IVSD (**e**) are shown. n = 5-8 independent animals. Representative photographs of the hearts of mice under TAC (**f**). Bars indicate 2 mm. HW/BW (**g**), HW/TL (**h**), LW/BW (**i**) and LW/TL (**j**) are shown. n = 5-8 independent animals. **k-o**, qRT-PCR analysis for expression of heart failure genes and pro-fibrosis genes in the hearts. n = 5-8 biologically independent samples. All values are means ± SEM. **c-e,** One-way ANOVA with Sidak's multiple comparisons test for comparison of groups. Two-tailed paired *t*-test between before and after treatment of the same group. **g-o**, One-way ANOVA with Sidak's multiple comparisons test. Numbers next to square brackets show significant *P* values.

Figure 1c

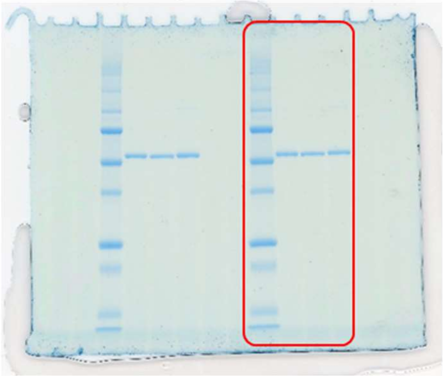

Supplementary Figure 11. Uncropped images of SDS-PAGE analysis.
